# Supplementary material for: Maternal health care services utilization and associated factors among pregnant women in Kersa district, Jimma zone, Southwest Ethiopia
Source: PLoS One. 2025 May 29;20(5):e0323977. doi: 10.1371/journal.pone.0323977 (PMC12121765; doi:10.1371/journal.pone.0323977)
Supplement: Attachment — Submitted filename: Response to reviewers.docx [file pone.0323977.s003.docx]

**Rebuttal Letter**

**To,**

**The Editor, Reviewers,**

**PLOS ONE**

**Date: February 15, 2025**

**Subject: Addressing Editor/ Reviewers Comments for PONE-D-24-46689**

**Dear Editor, Reviewers,**

Thank you for giving us the opportunity to submit a revised draft of the manuscript “**maternal health care services utilization in kersa district, Jimma zone, Southwest Ethiopia”** in the **Plos one** journal. We appreciate the time and effort that you and the reviewers dedicated to providing feedback on our manuscript and are grateful for the insightful comments on and valuable improvements to our paper. The authors have carefully considered the comments and tried our best to address every one of them. We hope the manuscript after careful revisions meets your high standards. The authors welcome further constructive comments if any. Below we are providing our response to the comments the point-by-point responses. The responses are highlighted in blue.

Thank you. Kind Regards,

Sincerely,

On behalf of co-author,

Tsedach Alemu Abebe

**Academic Editor:**

**1. Please ensure that your manuscript meets PLOS ONE's style requirements, including those for file naming. The PLOS ONE style templates can be found at**

[**https://journals.plos.org/plosone/s/file?id=wjVg/PLOSOne_formatting_sample_main_body.pdf**](https://journals.plos.org/plosone/s/file?id=wjVg/PLOSOne_formatting_sample_main_body.pdf) **and**

[**https://journals.plos.org/plosone/s/file?id=ba62/PLOSOne_formatting_sample_title_authors_affiliations.pdf**](https://journals.plos.org/plosone/s/file?id=ba62/PLOSOne_formatting_sample_title_authors_affiliations.pdf)

**Authors’ Response:** **we would like to thank the editor for all those useful comments. According to your suggestion, we have prepared the manuscript based on the PLOS ONE Journal requirements.** **We have updated the author affiliations so they match the requirements. We have added initials to the corresponding author after the email.**

**2. Please include captions for your Supporting Information files at the end of your manuscript, and update any in-text citations to match accordingly. Please see our Supporting Information guidelines for more information:** [**http://journals.plos.org/plosone/s/supporting-information**](http://journals.plos.org/plosone/s/supporting-information)**.**

**Authors’ Response: We have added a supporting information file or S1.Data (XLS) at the end of the manuscript.**

**Reviewer # 1:**

**General comments**

**Authors should review the manuscript and correct some grammatical errors and the manuscript needs basic editing throughout?**

**Authors’ Response: Thank you for your careful reading and your insights. We went through the entire manuscript to eliminate grammatical mistakes.**

**Topic: If the study includes a specific period (e.g., last one year), it would help make the topic more precise and indicate a timeframe for analysis.**

**Authors’ Response: We appreciate your suggestion to include the timeframe in the topic. While we do agree that stating the timeframe in the topic holds weight for everyone, however, this would lengthen the topic and take away from its clarity and generalizability. To address the issue, we incorporated the timeframe in the methodology section, where its inclusion flows more naturally next to the detailed description of the study design. We hope this solution sufficiently clarifies the temporal scope of the study. Please refer to lines # 148 to 149 of the methods section in the revised manuscript with track changes.**

**1. Abstract**

**A. Background**

**The statement notes that maternal mortality is a global concern and provides a general statistic (99% of preventable deaths in developing countries). It could be more precise by providing either recent data specific to Ethiopia or clarifying why Ethiopia’s situation is particularly concerning?**

**Authors’ Response: We thank you for this suggestion. We have added the data specific to Ethiopian context and revised the abstract section, background part. Please refer line # 34 to 35 in revised manuscript with track changes.**

**The phrase “maternal health care service utilization remains suboptimal” is somewhat vague. Specify in what way utilization is suboptimal?**

**Authors’ Response: Thank you for the comment. Correction has been made accordingly in the abstract section, background part. Please refer line # 36 to 37 in the revised manuscript with track changes.**

**B. Methods:**

**The sample of 562 pregnant women is specified, but the sampling method is not mentioned?**

**Authors’ Response: Thank you for this comment. Cluster sampling was used to select study participants. It has been mentioned in the abstract section, method part. Please refer line # 46 in the revised manuscript with track changes.**

**The statement "variables with a p-value of p≤0.05 were considered statistically significant" is clear, but it could be condensed by eliminating redundancy?**

**Authors’ Response: thank you for the comment. Correction has been made in the abstract section, method part. Please refer line # 53 to 54 in the revised manuscript with track changes.**

**C. Results:**

**The findings could be organized by outcome (e.g., ANC visits and institutional delivery) for clarity. Separating them into distinct sections may enhance readability.**

**Authors’ Response: thank you for suggestion. Correction has been made in the abstract section, result part. Please refer line # 56 to 58 in the revised manuscript with track changes.**

**Inconsistent terms (like "mothers" and "women") can disrupt the flow. Choose one term and stick to it for clarity?**

**Authors’ Response: Thank you for correction. We have chosen the word women to make clear the flow of the idea. Please refer revised manuscript with track changes all over the document.**

**D. Conclusion:**

**You conclude that “Maternal health care service utilization among pregnant women in the study area was found to be low” but what was your standard reference to say low?**

**Authors’ Response: We thank the reviewer for this comment. We have added the suggested comment to the manuscript abstract section, conclusion part. Please refer to lines # 71 in the revised manuscript with track changes.**
**2. Introduction:**

**The study could be strengthened by emphasizing the gap in local data and the importance of region-specific insights?**

**Authors’ Response: Thank you for suggestion. This has been updated and revised in the introduction section. Please refer to lines # 105 to 107 in the revised manuscript with track changes.**
**The disparities in ANC and institutional delivery rates between urban and rural women are repeated in multiple sentences. Condense this information for clarity?**

**Authors’ Response: Thank you for this comment. Correction has been made in the introduction section. Please refer to lines # 108 to 110 in the revised manuscript with track changes.**
**The benefits of maternal health services are stated multiple times in slightly different ways, which can be repetitive. Reorganization these points can enhance readability?**

**Authors’ Response: Thank you for your patient review. Correction has been made. Please refer the introduction section in the revised manuscript with track changes.**

**The trend in Ethiopia’s maternal mortality ratio is mentioned, but the comparison with SDG goals could be clarified more?**

**Authors’ Response: We thank you the reviewer for this comment. The data as suggested has been added in the introduction section. Please refer to lines # 85 to 87 in the revised manuscript with track changes.**
**3. Methods:**

**The description of the intervention could be clearer. It is also helpful to specify what the "standard care" entails for the control group?**

**Authors’ Response: Thank you for this suggestion. Correction has been made accordingly in method section, sampling procedure part. Please refer to lines # 187 to 188 in the revised manuscript with track changes.**
**Clarify whether informed consent was verbal or written?**

**Authors’ Response: Thank you for correction. Written informed consent was obtained from each woman. This has been corrected in the method section, sampling procedure part. Please refer to line # 181 in the revised manuscript with track changes.**

**You describe as your study participants for this study were pregnant women with 12 to 24 weeks of gestational age but why?**

**Authors’ Response: Again, we thank you the reviewer for this useful question. Pregnant women between 12 to 24 weeks of pregnancy were selected for this study to ensure that the intervention could be started early in pregnancy and give women enough time to complete the intervention. Recruiting women in the later weeks of pregnancy may not have given them enough time to complete the intervention within study timelines.**

**Needs to operationalize the following (low transportation costs, good road conditions**

**Authors’ Response: Thank you for the comment. The operational definition has been added in the method section, operational definition part. Please refer to lines # 232 to 235 in the revised manuscript with track changes.**

**4. Result:**

**The response rate is very low (82%). Did you use different methods to increase response rate?**

**Authors’ Response: Thank you for bringing this up, this is a very good point. Yes, different methods were used to increase the response rate. We tried to keep the survey questionnaire short and simple, the questions were arranged in a structured and easy method, and respondents' language was used, we explained briefly how respondents and their community would benefit from the survey results, we addressed each respondent by name and mentioned their relevance to the survey, the data was collected through face – to - face interviews, respondents assured confidentiality by removing all identifiers from the questionnaire and the right to refuse or withdraw at any time was respected, the respondents convenient time was chosen for the interview and their homes was revisited up to three times if respondents were not available at home.**

**Make the tables standardized?**

**Authors’ Response: Thank you for your very valuable comment. The tables has been updated accordingly. Please refer pages 11 to 17 in the revised manuscript with track changes.**

**Clarify the type of health workers seen and the location of ANC visits. For example, "nurses" might include various roles in different settings?**

**Authors’ Response: thank you the reviewer for this question. Antenatal care (ANC) in this study is defined according to the world Health Organization (WHO) as the health care provided to pregnant women by skilled healthcare professionals to ensure optimal health for both mother and baby during pregnancy. These skilled healthcare professionals, according to the World Health Organization (WHO), the International Confederation of Midwives (ICM), and the International Federation of Gynecology and Obstetrics (FIGO) definition, are accredited healthcare professionals such as midwives, doctors, and nurses who are trained to manage normal pregnancies, deliveries and postpartum care, as well as recognize and refer complications. In the Ethiopian context, skilled healthcare professionals include midwives, doctors, health officers, and nurses working in government hospitals, government health centers, private hospitals and private clinics. This study focused on women receiving ANC services at government hospitals or health centers, as these facilities are more accessible in rural areas where the population faces financial barriers to private health facilities. Government facilities offer free maternal health services, including ANC and delivery, making them the preferred choice for many. Health posts were excluded from this study as the health workers in those facilities, known as health extension workers in Ethiopia, do not meet the criteria of skilled health care professionals as defined above.**

**5. Discussions
Unclear and vast discussions.**

**Authors’ Response: thank you the comment. The discussion has been revised. Please refer to the discussion section page 18 to 21 in the revised manuscript with track changes.**

**The discussion tries to compare and contrast the findings from other research findings which are not similar with the current study (in the context of study population, setting…**

**Authors’ Response: thank you for the comment. Correction has been made. Please refer the discussion section page 18 to 21 in the revised manuscript with track changes.**

**Justification of the discrepancy is not as such strong? Please make it clear and precise**

**Authors’ Response: thank you for the comment. Correction has been made. Please refer to the discussion section page 18 to 21 in revised manuscript with track changes.**

**6. Recommendations**

**Recommendation should be based on your findings?**

**Authors’ Response: Thank you for suggestion. Correction has been made. Please refer to recommendation section page 21 to 22, lines # 494 to 499 in the revised manuscript with track changes.**

**Your limitations and strengths are not rational?**

**Authors’ Response: Thank you for the comment. Correction has been made. Please refer to limitation and strength part page 22, lines # 517 to 521 in the revised manuscript with track changes.**

**Reviewer # 2:** **My Comments to authors**

**Thank you very much for giving me the opportunity to review this paper. In general, it requires serious amendments. Here below are my comments to improve the paper:**

**Abstract**

**1. Key words should be revised-based MeSH terms. Are Kersa District and Jimma Zone important as key terms?**

**Authors’ Response: Thank you for the comment. Correction has been made in abstract key words based on suggestion. Please refer line # 76 in revised manuscript with track changes.**

**2. The result in the abstract needs some modification in such a way that it is clear for all readers.**

**Authors’ Response: Thank you for the comment. Correction has been made. Please refer the abstract section, result part in the revised manuscript with track changes.**

**3. Conclusion and recommendation not support the finding of the article.**

**Authors’ Response: Thank you for the comment. Correction has been made based on suggestion. Please refer the abstract section, conclusion and recommendation part, lines # 70 to 75 the revised manuscript with track changes.**

**4. As written in the submission guidelines of PLOS ONE, you have to include the line number to make easy reviewing.**
**Authors’ Response: Thank you for the comment. Line numbers have been included. Please refer revised manuscript with track changes.**

**Introduction**

1. **first two sentences need citation**

**Authors’ Response: Thank you for the comment. Correction has been made based on suggestion. Please refer line # 79 to 81 in the introduction section of revised manuscript with track changes.**

2. **You have started the sentence like ‘99% of mortality...’ Please edit for the grammar and spacing**

**Authors’ Response: We thank you the reviewer for this comment. Correction has been made accordingly. Please refer line # 81 in the introduction section in revised manuscript with track changes.**

3. **Please be consistent in the reference citation. Some of the citations are after full stop and others are before full stop (newborn.7 vs visits 8.)**

**Authors’ Response: Thank you for the comment. Correction has been made accordingly. Please refer revised manuscript with track changes in the whole document.**

4. **This part needs extensive grammar and citation editing and conceptual maturation of the problem.
   Authors’ Response: Again, we thank you the reviewer very much for this useful comment. Correction has been made based on suggestion. Please refer revised manuscript with track changes the whole document.**

**Method**

1. **Your study was conducted from October 23 to December 17, 2020. It was almost five years ago. Why are you late to submit the article for publication? Data five years ago is not reliable for publication.**

**Authors’ Response: Thank you for this valuable question. The late submission of the article for publication was caused by the country’s political instability, internal conflicts and COVID-19 interrupted data collection in the community. Moreover, the lack of access to electricity and the internet due to internal conflicts made it difficult to search for literature and enter data. In addition, the corresponding author experienced a prolonged period of severe illness which affected the preparation of the manuscript.**

**What are the best practice standards for maintaining research records after completion for publication?**

**Authors’ Response: Thank you for this question.**

- **Keep research records for a minimum of 5-10 years.**
- **Store data in pass word protected digital repositories, fireproof locked cabinets for physical documents.**
- **Maintain at least two backup copies in different locations. eg. External hard drive.**
- **Deposit datasets in trusted repositories. Eg. Institutional databases.**
- **Remove any personal identifiers from datasets.**
- **Clearly document variables, methodologies, and any modifications made during the research process.**
- **Use structured folders, labeled files, and detailed descriptions for easy retrieval.**
- **Limit access to authorized individuals only.**
- **Adhere to data-sharing policies mandated by journals and funding agencies.**
- **Changing published articles to policy briefs.**

2. **The sample size determination is good. But how did you come up with the design effect of 2? How can it be 2? Why not other numbers? You have to show.**

**Authors’ Response: Thank you for pointing this out. The design effect for this study, due to cluster randomization was calculated using intraclass correlation coefficient (ICC) of 0.03; which was taken from a previous similar study, and the average cluster size (m) = 34.**

**Design effect= 1+ (m-1) × ICC.**

**Design effect = 1+ (34-1) × 0.03 = 1.99 ~ 2**

**This has been updated in the method section, the sample size calculation part. Please refer to sample size calculation, line # 164 to 165 in the revised manuscript with track changes.**

3. **You determined the sample size of an estimated 686 respondents during sample size calculation, but the study was conducted among 562 pregnant women. Why does this disparity occur (686 -562 = 124)?**

**Authors’ Response: Thank you for bringing this up, this is a very good point. The discrepancy (686-562=124) occurred because this baseline study assessed the utilization of previous institutional delivery and antenatal care services in previous pregnancy. However, 124 participants were primigravida (experiencing their first pregnancy) and had not used these services before. Therefore, they were excluded from this baseline study. The remaining 562 multigravida pregnant women, who had previous use of these services, were included as study participants for this study.**

**Sampling procedure not clear,**

**Authors’ Response: Thank you the reviewer for this comment. The sampling procedure has been revised. Please refer to the sampling procedure part page 6 to 7, lines # 169 to 188 in the revised manuscript with track changes.**

**It is for interventional study or for your big project not for baseline study. Please clarify it.**

**Authors’ Response: Thank you for this comment. This is a baseline study for an interventional study. The baseline study was conducted first, followed by the implementation of the intervention. Please refer to sample size calculation line # 154 in revised manuscript with track changes.**

**5. Figure 1 is a trail flow diagram. Why is it important in a baseline study?**

**Authors’ Response: Thank you for this comment. Figure 1 has been removed. Please refer page 7 in the revised manuscript with track changes.**

**6. How do you measure institutional delivery services in this baseline study? Your study participants were pregnant mothers. Are you asking previous delivery service utilization?**

**Authors’ Response: We thank you the reviewer for this question. Yes, women were asked about their use of institutional delivery services during their previous childbirth. For this baseline study, institutional delivery service utilization was measured from previous institutional delivery service use.**

**7. Please clearly operationalize your outcome variable maternal health care services (antenatal care and institutional delivery services utilization).**

**Authors’ Response: Thank you for this comment. Correction has been made on operational definition of the outcome variables. Please refer Page 7 to 8, lines # 201 to 210 in the revised manuscript with track changes.**

**Result**

**1. What is the importance of a detailed description of knowledge on obstetric danger signs among pregnant women? You can compute it and use it as one predictor variable for the outcome variable.**

**Authors’ Response: Thank you for your careful reading and your insights. Detailed description of knowledge on obstetric danger signs has been removed in the result part of the manuscript. Please refer to result section page 10 to 17 in revised manuscript with track changes.**

**2. Why do you omit government employee from the category of both wife and husband occupation?**

**Authors’ Response: Thank you for this question. There were no study participants who were government employees. This may be due to the rural nature of the study area.**

**3. How do you compute the wealth index? Have you done PCA for it?**

**Authors’ Response: Thank you for this question. Yes PCA has done. In this study, Wealth index was calculated using the following ownership of household resources: Type of floor, wall and roof material that the house-made, number of rooms household have, separate kitchen rooms household have, household source of drinking water and water source for cooking, toilet facility, type of fuel use for cooking, ownership of agricultural land, homes and livestock such as cattle, sheep, goat, milk cows, oxen, horses, donkey, chicken, and small equipment such as landline phone, mobile phone, radio, television, electricity, bicycle, motorcycle and car. Six principal components with eigenvalues greater than one were summed to obtain wealth index values after principal component analysis (PCA) was run. The resulting index was then divided into three categories presenting first quantile (poor), second quantile (medium) and third quantile (rich). Please refer to method section, operational definition part pages 8, lines # 216 to 277 in the revised manuscript with track changes.**

**Discussion**

1. **You have to modify introductory paragraph for discussion**

**Authors’ Response: Thank you for the comment. Correction has been made. Please refer to discussion section on page 18 in the revised manuscript with track changes.**

2. **There is redundancy on at the beginning of discussion**

**Authors’ Response: Thank you for the comment. Correction has been made. Please refer to discussion section page 18 in the revised manuscript with track changes.**

3. **Why not include the value of the predictors during discussion? Instead of saying, ‘Women with private employee husbands were more likely utilize antenatal care services than farmer husbands.’ You can write as women with private employee husbands were two times more likely utilize antenatal care services than farmer husbands.**

**Authors’ Response: Thank you for the comment. Correction has been made. Please refer to discussion section page 19 to 21 in the revised manuscript with track changes.**

4. **In general, the discussion part is somewhat good but needs few modification.**

**Authors’ Response: We appreciate the reviewer for the positive assessment of our work. The discussion has been revised. Please refer to discussion section page 18 to 21 in the revised manuscript with track changes.**

5. **Can a researcher be an author and acknowledged at the same time? Please differentiate acknowledgement versus authorship. In your study, the co-author is also acknowledged. Why?**

**Authors’ Response: Again, we thank you the reviewer very much for this useful correction. The co-author in the acknowledgment part has been removed. Please refer to acknowledgement part page 22, line # 522 to 525.**

6. **6 Please include URL for some references like R1, R2 and R3**

**Authors’ Response: Thank you for the comment. URL has been added to some of the references. Please refer to reference part pages 23 to 28.**
