# Supplementary material for: Maternal health care services utilization and associated factors among pregnant women in Kersa district, Jimma zone, Southwest Ethiopia
Source: PLoS One. 2025 May 29;20(5):e0323977. doi: 10.1371/journal.pone.0323977 (PMC12121765; doi:10.1371/journal.pone.0323977)
Supplement: Attachment — Submitted filename: Response_to_reviewers_auresp_2.docx [file pone.0323977.s004.docx]

**Rebuttal Letter**

**To,**

**The Editor, Reviewers,**

**PLOS ONE**

**Date: March 26, 2025**

**Subject: Addressing Editor/ Reviewers Comments for PONE-D-24-46689**

**Dear Editor, Reviewers,**

Thank you for giving us the opportunity to submit a revised draft of the manuscript “**maternal health care services utilization in kersa district, Jimma zone, Southwest Ethiopia”** in the **Plos one** journal. We appreciate the time and effort that you and the reviewers dedicated to providing feedback on our manuscript and are grateful for the insightful comments and valuable improvements to our paper. The authors have carefully considered the comments and tried our best to address every one of them. We hope the manuscript after careful revisions meets your high standards. The authors welcome further constructive comments if any. Below we are providing our response to the comments the point-by-point responses. The responses are highlighted in blue.

Thank you. Kind Regards,

Sincerely,

On behalf of co-author,

Tsedach Alemu Abebe

**Academic Editor:**

**Journal Requirements:

Please review your reference list to ensure that it is complete and correct. If you have cited papers that have been retracted, please include the rationale for doing so in the manuscript text, or remove these references and replace them with relevant current references. Any changes to the reference list should be mentioned in the rebuttal letter that accompanies your revised manuscript. If you need to cite a retracted article, indicate the article’s retracted status in the References list and also include a citation and full reference for the retraction notice.**

**Authors’ Response: We thank you the editor for this comment. The references have undergone numerous checks for problems.**

- **The references previously listed as references 6 and 13 have been removed from the manuscript.**
- **A new reference or reference 12 has been added to the manuscript.**
- **No changes were made to the remaining references. Please refer to reference 12 in the revised manuscript with track changes.**

**A previous reference listed as reference 6 has been removed from the manuscript: (Graham H, Bell J, Bullough C. Can Skilled Attendance at Delivery Reduce Maternal Mortality in Developing Countries? Safe Mother Strateg A Rev Evid [Internet]. 2001; Studies in: 1-28).**

**A previous reference listed as reference 13 has been removed from the manuscript: (Bista S. Utilization of Maternal Health Care Services in Province 2, Nepal. Nepal. J Popul Dev. 2022; 3(1):141-50).**

**A new reference (reference 12) has been added to the manuscript: (Adedokun ST, Uthman OA, Bisiriyu LA. Determinants of partial and adequate maternal health services utilization in Nigeria: analysis of cross-sectional survey: BMC Pregnancy Childbirth. 2023; 23(457):1-11.)**

**Review Comments to the Author**
**Reviewer #3:**

**General Comment:
The clean copy of the manuscript does not appear to have been revised in accordance with the reviewers’ comments.**

**Introduction:**

**1. Line 109: When reporting statistics about the use of skilled birth attendance by urban versus rural women, it is essential to include the year of the investigation Authors’ Response: Thank you the reviewer for this comment. The year of the investigation has been added. Please refer to lines # 91 to 92 in the revised manuscript with track changes.**

**2. Please provide information on the national guidelines for antenatal care (ANC) in Ethiopia. For example:**

**o At what gestational age do women typically begin ANC?**

**Authors’ Response: We thank you the reviewer for this comment. The Ethiopian national guide lines for antenatal care recommends ANC begins in the first trimester of pregnancy (up to 12 weeks of gestational age).**

**o How many ANC visits are recommended?**

**Authors’ Response: The guideline recommends a minimum of eight visits for all pregnant women who do not have any pregnancy-associated complications. For those pregnant women with identified problems, additional visits may be scheduled as necessary.**

**o Who is responsible for providing ANC services?**

- **Authors’ Response: According to the Ethiopian antenatal care guideline, the Health professionals who are responsible for ANC services provision are:-**
- **Obstetrician & gynecologist**
- **Integrated emergency surgical officer**
- **Midwives**
- **Health officers**
- **Nurses**
- **Doctors**
- **Level IV health extension workers**
- **Laboratory professionals and**
- **Radiologists**
- **For further explanation, ANC guideline of Ethiopia by level of health facility and type of health worker providing ANC are described in the following table. Ethiopia's current national guidelines for antenatal care (ANC) were developed in 2022 as per the World Health Organization (WHO) ANC recommendations adaption toolkit, referring to the WHO 2016 comprehensive recommendations on ANC for pregnant women and adolescent girls. Ethiopia is replacing the previous four-visit Focused Antenatal Care (FANC) model with the new ANC eight-visit model. To reduce perinatal mortality and improve the pregnancy experience of women, a minimum of eight visits is required. For those pregnant women with identified problems, additional visits may be scheduled as necessary. The first visit is recommended in the first trimester (up to 12 weeks of gestational age).**

| **Level of health facility** | **Type of health worker**  **Providing ANC** | **ANC services** |
| --- | --- | --- |
| **Comprehensive health posts with Laboratory facilities** | - **Midwives,** - **Health officers** - **Nurses, and** - **Health extension workers** | - **Perform ANC contacts for pregnant women with no identified problem.** - **Take history and perform physical examination.** - **Identify and link pregnant woman to health facilities.** |
| **Health center** | - **Midwives** - **Health officers** - **Nurses** - **Integrated emergency surgical officer** - **Doctors and, laboratory professionals** | - **Perform 8 contacts of ANC according to the guide line.** - **Take history and perform physical examination.** - **Perform laboratory tests (hemoglobin/hematocrit, blood group and RH, indirect Coomb’s for Rh positive pregnant women, urine analysis (dipstick for protein), urine gram stain, test for HIV, syphilis and hepatitis B virus.** - **Perform ultrasound.** - **Administer prophylaxis (iron, folate, calcium, tetanus-diphtheria vaccination, deworming after 1^st^ trimester and anti-D for Rh negative pregnant women** - **Perform on lifestyle modification, danger signs and symptoms, birth preparedness and complication readiness and optimal maternal nutrition.** - **Provide treatment for illness such as bacteriuria, UTI, malaria, HIV, syphilis, headache and others.** - **Provide emergency care and refer to the hospital for malpresentation.** |
| **Hospital** | - **Obstetrician & gynecologist** - **Integrated emergency surgical officer** - **Midwives** - **Health officers** - **Nurses** - **Doctors** - **Laboratory professionals and** - **Radiologists** | **In addition to health center services**   - **Manage any pregnancy related complications such as severe anemia, hypertension, diabetes mellitus, any bleeding related to pregnancy, severe infection and other chronic illness.** - **Perform antepartum fetal surveillance** - **Follow and manage multiple pregnancy and hyperemesis** |

**Methods:**
**1. Please clarify why women with a gestational age of 12–24 weeks were specifically recruited for this study.**

**Authors’ Response: Thank you for this question. As this study is the baseline assessment for a big interventional study, Pregnant women between 12 to 24 weeks of pregnancy were selected to ensure that the intervention could be started early in pregnancy and give women enough time to complete the intervention. Recruiting women in the later weeks of pregnancy may not have given them enough time to complete the intervention within study timelines.**

**2. Line 170: Since this is a cross-sectional study, randomization was not applicable. Instead, random selection was used. Please revise the text to reflect this distinction clearly.**

**Authors’ Response: Thank you the reviewer for this useful comment. We have corrected it based on suggestions. Please refer to line # 155 in the revised manuscript with track changes.**

**3. I recommend that the authors clearly separate the description of this cross-sectional study from any interventional study. The current writing style may confuse readers about the study design.**

**Authors’ Response: We thank you the reviewer for this useful comment. A correction has been made based on the suggestion. Please refer to page 6, lines # 151 to 178 in the revised manuscript with track changes.**

**Results:**
**1. Table 1: Please include the units of measurement for each variable to enhance clarity.**

**Authors’ Response: We appreciate your suggestion to include units of measurement for each variable. While we do agree that including the units of measurement for each variable enhances clarity, to the best of our knowledge as researchers, units of measurement could not be identified for variables except age. Therefore, we have added units of measurement only age. Please refer to Table 1 in the revised manuscript with track changes.**

**2. Table 2: The term “experienced pregnancy complications” is unclear. Please define what specific complications were included in this category.**

**Authors’ Response: Thank you for this comment. Pregnancy complications included in this study were: severe vaginal bleeding, severe headache, blurred vision, convulsion, swollen hands/face, high fever, loss of consciousness, difficulty breathing, severe weakness, severe abdominal pain, fetal movement problems (reduced or increased fetal movements), and water breaks without labor.**

**3. Regarding “severe bleeding,” was this referring to postpartum hemorrhage (PPH)? If so, please specify.**

**Authors’ Response: We thank the reviewer for this comment. The term “severe bleeding” in Table 2 does not refer to postpartum hemorrhage; Instead, it refers to Severe bleeding during pregnancy. We have corrected to specify it as severe vaginal bleeding during pregnancy. Please refer to Table 2 in the revised manuscript with track changes.**

**4. Table 3: For “Home delivery,” please indicate whether a health provider (e.g., a midwife) was present during the delivery.**

**Authors’ Response: We thank you the reviewer for this comment. The primary outcome variables in this study were maternal health care services utilization, specifically antenatal care, and institutional delivery services utilization. Skilled birth attendance was not the primary focus of this study. Therefore, the presence of skilled providers was only assessed for institutional deliveries. No assessment was made regarding the presence of skilled providers for home deliveries.**

**5. Table 4: Please specify the factors for which the adjusted odds ratios (ORs) were calculated.**

**Authors’ Response: Thank you for the comment. Factors such as occupation of wife, family size, wealth index, health service quality, distance to health facility, adequate transport available, age of women, occupation of husband, knowledge of danger signs during pregnancy, road conditions and transportation cost were adjusted odds ratios calculated.**

**Discussion:**

**1. One of the authors’ recommendations is to “increase household income.” This recommendation falls outside the scope of the Ministry of Health’s decision-making authority and would require significant government investment. Please consider revising this recommendation to focus on actionable steps within the health sector’s capacity.**

**Authors’ Response: We appreciate your suggestion that the recommendation not falls outside the scope of the Ministry of Health’s decision-making authority. While we do agree with this concern, this study identified a significant association between wealth index and the utilization of institutional delivery and antenatal care services (please refer to Table 4 & Table 5). Based on this finding, this study recommended an increase in household income. Moreover, evidence from global studies suggests that a multisectoral approach is more effective than a single-sector approach in problem-solving. Therefore, problem-solving does not rest solely on the health sector; other relevant sectors including government bodies and additional stakeholders should also take part in problem-solving.**
