# Supplementary material for: Maternal health care services utilization and associated factors among pregnant women in Kersa district, Jimma zone, Southwest Ethiopia
Source: PLoS One. 2025 May 29;20(5):e0323977. doi: 10.1371/journal.pone.0323977 (PMC12121765; doi:10.1371/journal.pone.0323977)
Supplement: Attachment — Submitted filename: Response_to_reviewers_auresp_3.docx [file pone.0323977.s005.docx]

**Rebuttal Letter**

**To,**

**The Editor, Reviewers,**

**PLOS ONE**

**Date: April 10, 2025**

**Subject: Addressing Editor/ Reviewers Comments for PONE-D-24-46689**

**Dear Editor, Reviewers,**

Thank you for giving us the opportunity to submit a revised draft of the manuscript “**maternal health care services utilization in kersa district, Jimma zone, Southwest Ethiopia”** in the **Plos one** journal. We appreciate the time and effort that you and the reviewers dedicated to providing feedback on our manuscript and are grateful for the insightful comments and valuable improvements to our paper. The authors have carefully considered the comments and tried our best to address every one of them. We hope the manuscript after careful revisions meets your high standards. The authors welcome further constructive comments if any. Below we are providing our response to the comments the point-by-point responses. The responses are highlighted in blue.

Thank you. Kind Regards,

Sincerely,

On behalf of co-author,

Tsedach Alemu Abebe

**Academic Editor:**

Journal Requirements:

Please review your reference list to ensure that it is complete and correct. If you have cited papers that have been retracted, please include the rationale for doing so in the manuscript text, or remove these references and replace them with relevant current references. Any changes to the reference list should be mentioned in the rebuttal letter that accompanies your revised manuscript. If you need to cite a retracted article, indicate the article’s retracted status in the References list and also include a citation and full reference for the retraction notice.

**Authors’ Response: Thank you the editor for this comment. We have checked the references several times to ensure their accuracy. Based on our repeated checks, we confirm that all references are accurate and complete, and there are no retracted papers.**

**Reviewer #3:**

Thanks to the authors as they responded my previous comments comprehensively. However, the responses provided by the article's authors to each section of the introduction, methodology, and results were not incorporated into the paper and remained unavailable for review."

**Authors’ Response: Thank you the reviewer for this comment. Our responses to your previous comments are available in the revised manuscript with track changes.**

**Introduction section**

**Our response to your previous comment on the introduction section is available in lines # 90 to 91 in the revised manuscript with track changes.**

**Methodology section**

**Our response to your previous comments on the methodology section are available in lines # 127 to 177 in the revised manuscript with track changes.**

**Result section**

**Our response to your previous comments in the result section are available in Table 1 and Table 2.**

**Please refer to the revised manuscript with track changes for more clarification.**

**Thank you.**
